# From medicine cabinets to ecosystems: a Europe-wide assessment of household pharmaceutical waste disposal practices

**DOI:** 10.3389/fphar.2026.1788038

**Published:** 2026-04-28

**Authors:** Przemyslaw Kardas, Tamas Agh, Disposal Collaborators, Margaret Bermingham

**Affiliations:** 1 Medication Adherence Research Center, Department of Family Medicine, Medical University of Lodz, Lodz, Poland; 2 Medication Adherence Research Group, Center for Health Technology Assessment and Pharmacoeconomic Research, University of Pécs, Pécs, Hungary

**Keywords:** environmental impact, Europe, extended producer responsibility, health policy, household pharmaceutical waste, medication optimisation, medicine disposal, pharmaceutical pollution

## Abstract

**Background:**

Unused and expired household medicines contribute to environmental pollution, avoidable healthcare costs, and public health risks, including antimicrobial resistance. Although regulatory frameworks for pharmaceutical waste disposal exist across Europe, real-world practices remain poorly characterised and unevenly implemented. Comprehensive, contemporary evidence on how household medication disposal systems operate across European countries is lacking.

**Methods:**

A Europe-wide, cross-sectional online expert survey was conducted from 17 March to 15 October 2025. Experts in healthcare, pharmacy, regulation, and health policy provided structured information on national legislation, collection systems, disposal practices, public awareness activities, and economic arrangements for unused or expired household medicines. Data were consolidated at the country level and analysed descriptively.

**Results:**

Forty valid responses were received from experts across 35 European countries. Dedicated legislation for household pharmaceutical waste was reported in 30 countries, while five lacked specific legal provisions and three reported no formal collection system. Pharmacy-based take-back schemes operated in 31 countries, yet their organisation, funding, and effectiveness varied widely. In eight countries, unused medicines were commonly discarded in household waste. Annual collection rates varied from 12.4 to over 200 g/capita. Extended producer responsibility schemes were reported in six countries and were associated with higher collection volumes and comprehensive national coverage. Public awareness campaigns were reported in 22 countries, but their scope, visibility, and evaluation were inconsistent. Systematic monitoring data were unavailable in most countries, and only seven reported reimbursement mechanisms for pharmacies collecting medicines.

**Conclusion:**

Household medication disposal practices across Europe remain highly heterogeneous, reflecting persistent gaps between policy intent and implementation. Inconsistent legislation, limited financing, variable public awareness, and weak monitoring undermine system performance. Coordinated European action is needed to harmonise standards, strengthen financing, and promote environmentally safe disposal of unused and expired household medicines.

## Introduction

Atop longstanding demographic and economic challenges, healthcare systems are now facing growing pressure to meet rising environmental expectations. As major consumers of energy, materials, and pharmaceuticals, they contribute substantially to environmental degradation; being responsible for more than 5% of global CO_2_ emissions (Keller et al., 2021). Within this broader environmental footprint, medication use plays a particularly important role. Pharmacotherapy remains a cornerstone of modern medicine, essential for the prevention, treatment, and management of a wide range of acute and long-term conditions. Yet it also entails risks for both patients and the environment, with pharmaceuticals accounting for approximately 12% of healthcare-related carbon emissions (Keller et al., 2021). These risks become particularly evident in the case of unused or expired household medications. Ideally, the generation of such waste would be minimised. This can be achieved through broader implementation of medication optimisation and deprescribing, which have been shown to reduce potentially inappropriate prescriptions and overall medication burden (Bloomfield et al., 2020). Improving medication adherence may also help alleviate this burden, as poor adherence contributes significantly to the accumulation of unused medicines (Kardas and Agh, 2025) (van den Bemt et al., 2025). However, even the most effective preventive strategies cannot entirely eliminate the problem. Unused or expired drugs will continue to arise due to therapy changes necessitated by ineffectiveness or adverse events, or as a consequence of patient death (Bekker et al., 2018). A certain volume of medicinal waste is therefore unavoidable. In this study, such waste is referred to as ‘household pharmaceutical waste’, defined as unused or expired medicines remaining in households after intended use by patients.

In this context, safe and effective disposal systems are essential. Although current legislation provides a regulatory framework for the disposal of unused or expired medicinal products, including the overarching European Directive 2001/83/EC, evidence suggests that existing systems do not function optimally, and the management of household pharmaceutical waste remains insufficiently organised. Globally, the predominant method for disposing of unused medications is still through household waste or sewage (Nepal et al., 2020). However, this practice is also widespread in high-income countries, including several European ones (Jafarzadeh et al., 2021; Organisation for Economic Co-operation and Development, 2022; Rogowska and Zimmermann, 2022; Paut et al., 2017; Rogowska et al., 2019). Similar scenarios are observed even in countries with established take-back programmes, indicating that these measures alone are insufficiently effective (Rogowska and Zimmermann, 2022).

The consequences of this situation are profound. Even when disposed of properly, unused or expired drugs are typically destroyed through high-temperature incineration, resulting in substantial carbon emissions and, in some cases, toxic pollution (Blackburn et al., 2024). However, it is mainly improper disposal practices that pose a significant threat to both terrestrial and aquatic ecosystems. Medicinal residues, including antibiotics, analgesics, hormones, and non-steroidal anti-inflammatory drugs, are now detectable in surface waters, sediments, and soils across diverse geographical regions (Cussans et al., 2021; Luo et al., 2021). A recent report identified around 60 different active pharmaceutical ingredients in Scottish rivers, lakes, and wastewater, highlighting the scale of environmental contamination (Helwig et al., 2022). These substances pose serious ecological and public health risks. Improper disposal of unused household medicines also has a well-recognised contribution to antimicrobial resistance: the presence of antibiotics in ecosystems significantly contributes to the development and spread of antimicrobial resistance (Cussans et al., 2021; European Health Management Association, 2022; Luo et al., 2021; Hamilton et al., 2024; United Nations Environment Programme, 2023). Beyond microbial threats, adverse effects on wildlife have been observed, including fish mortality and endocrine disruption leading to intersex conditions in riverine species (Cussans et al., 2021; Luo et al., 2021; National Institute for Health and Care Excellence, 2015). Finally, improper medication disposal leads to avoidable healthcare costs and preventable injuries, including accidental poisonings (Nepal et al., 2020; Kaya and Genç, 2025; Insani et al., 2020). Thus, the consequences of improper disposal span human, animal, and environmental health, reflecting the holistic One Health framework (Thornber et al., 2022; Wilkinson et al., 2022; Richie et al., 2023).

In light of these challenges, there is an urgent need to develop and optimise strategies that ensure environmentally safe pathways for medication disposal. Previous research on household pharmaceutical waste disposal has relied mainly on narrative reviews, single-country studies, or regionally restricted project reports (Organisation for Economic Co-operation and Development, 2022; Rogowska and Zimmermann, 2022; Nepal et al., 2020; Paut et al., 2017; Mehtonen et al., 2020; Kanyari et al., 2024). While these studies have provided valuable insights into some key aspects of pharmaceutical waste management and its environmental implications, systematic and up-to-date evidence on how disposal systems operate across Europe remains limited. In particular, a pan-European overview integrating legislation, organisational models, collection practices, public awareness activities, and economic arrangements has not previously been available. Therefore, the aim of this study was to provide a comprehensive overview of current practices for disposing of unused or expired household medicines across all European countries.

## Methods

In this Europe-wide, cross-sectional online expert survey, conducted from 17 March to 15 October 2025, we invited experts in healthcare, health policy, pharmaceutical regulation, and related fields who possessed extensive knowledge of the management of unused and expired medicines. Participants were asked to provide information on both the theoretical framework and practical implementation of medicine disposal practices within their primary country of professional activity. The survey was organised by the Medical University of Lodz and, in accordance with the policy of the University’s Ethical Committee, did not require formal ethical approval, as no sensitive personal data were collected. This study was reported according to the Checklist for Reporting Results of Internet E-Surveys (CHERRIES) (Eysenbach, 2004).

### Questionnaire

In order to collect national experts’ feedback in a systematic way, a dedicated questionnaire was developed based on the authors’ expertise and a review of the existing literature. The questionnaire consisted of items covering seven domains at the national level: (i) respondents’ professional profile; (ii) legislative frameworks governing the collection of unused or expired household medications; (iii) characteristics of collection programmes; (iv) practices for medication collection; (v) availability of awareness campaigns; (vi) economic dimensions of unused or expired household drug disposal; and (vii) additional information relevant to the study subject. Wherever applicable, respondents were invited to provide references to external resources and scientific publications, which are subsequently cited in the Results section.

Prior to launch, a pilot survey was conducted in February 2025 among selected European experts. Based on the feedback from the pilot, a revised questionnaire was validated by the core study group and approved (Supplementary Material 1). The final version was made available online via the SurveyMonkey cloud-based software (www.surveymonkey.com) for data collection.

### Participants

The survey employed a targeted, invitation-based recruitment strategy. Potential participants were identified among experts affiliated with European scientific networks, including members of the COST Action ENABLE (‘European Network to Advance Best practices and technoLogy on medication adherencE’, CA19132), European Drug Utilisation Research Group (EuroDURG), and the European Society of Clinical Pharmacy (ESCP). Experts were purposively selected to ensure relevant domain expertise in pharmaceutical policy, medication disposal systems, public health, regulatory affairs, or environmental health. Eligibility criteria included at least one of the following: (i) professional involvement in national pharmaceutical regulation or policy-making, (ii) academic research in pharmacoepidemiology, pharmaceutical waste management, or related disciplines, or (iii) leadership roles in professional or governmental bodies responsible for medication disposal systems. Invitations were sent via email, containing a brief study overview, the objectives of the survey, and a secure link to the online questionnaire. A follow-up reminder was sent approximately 2 weeks after the initial invitation to maximize the response rates. In case of lack of response, other experts were sought to ensure European-wide coverage. Because recruitment followed an iterative, network-based approach, the exact number of experts who received the invitation could not be precisely determined and a formal response rate was therefore not calculated.

Participants were encouraged to provide an accurate reflection of the real-world situation in their countries, rather than an idealized version reported by official sources. Participation was fully voluntary, no personal or sensitive data were collected, and participants’ consent was implied by completion of the survey.

### Data analysis

Data collected via the online survey system were downloaded into a database and systematically reviewed. Responses provided under ‘other’ categories were carefully examined and, where appropriate, recoded to match one of the standard answer options. Multiple responses from a single country were compared and reconciled. In case of discrepancies, clarification was sought through follow-up communication. Where necessary, responses were cross-checked against publicly available legal or regulatory documents. If inconsistencies could not be fully resolved, the most conservative interpretation was adopted, or variability was explicitly acknowledged in the dataset. This approach aimed to enhance data reliability while preserving transparency. At the end of this process, a single, consolidated profile was created for each country included in the study.

The country-specific data were then summarized descriptively, providing detailed insights into each country’s legislative frameworks, collection programmes, practices, awareness campaigns, and economic dimensions. Each domain was analysed individually, highlighting the availability of information and capturing specific national circumstances and trends. While this approach effectively illustrates variations across countries within each domain, no direct, one-to-one comparisons between countries were performed, ensuring that the study focuses on descriptive analysis rather than comparative metrics. The approach reflected the exploratory nature of the study and the objective of mapping cross-national differences in medication disposal systems. Given the relatively small number of participating countries and the predominantly categorical structure of the collected information, formal comparative statistical modelling was not considered methodologically appropriate. The descriptive approach allowed transparent presentation of system-level differences without introducing assumptions that could not be robustly supported by the available data.

For cross-country comparisons, categorical variables were compared using Fisher’s exact test, while continuous or ordinal variables were analysed using the Wilcoxon rank-sum test. Effect sizes for associations were quantified using Cramér’s V. All statistical analyses were conducted using R software (version 4.5.1).

## Results

### Study participants and geographic coverage

The survey received 40 valid responses, some of which were submitted jointly by two or three experts, from 35 countries, covering all but one EU member state (Malta), as well as Albania, Bosnia and Herzegovina, Iceland, Montenegro, North Macedonia, Norway, Switzerland, Türkiye, and the United Kingdom. Full country level dataset has been provided in Supplementary Material 2.

For consistency, demographic data refer to the primary respondent identified for each response. Most primary respondents had primary expertise in healthcare or pharmacy (31/40), over 10 years of relevant professional experience (33/40), and were primarily affiliated with academic institutions (30/40).

### Legislative background

Most countries reported having national legislation that specifically addresses the collection of unused or expired household medications. These frameworks include National Waste Acts or equivalent regulations (20/35), specific decrees or laws classifying unused or expired household medications as hazardous waste and regulating their management (6/35), and, less frequently, obligations established under Extended Producer Responsibility (EPR) schemes, which make pharmaceutical manufactures financially responsible for the collection and destruction of unused medicines, or municipal-level rules for waste handling. However, five countries - Greece, Luxembourg, Montenegro, North Macedonia, and the United Kingdom - reported the absence of legislation specifically dedicated to this issue.

In countries where dedicated collection systems operate, patients can generally return unused or expired medications anonymously. Notably, Luxembourg and Slovenia reported exceptions to this practice, indicating that anonymity may not be fully ensured. Penalties for improper disposal of unused or expired medications (e.g., flushing, burning, or discarding in household waste) were reported in nine of 35 countries. Reported fines varied substantially, ranging from €95 in the Netherlands, €150–500 in Bulgaria, and €360 in Austria, to as high as €1,500 in Slovakia. However, according to respondents, such penalties are rarely enforced in practice.

### Collection systems for unused and expired household drugs

Dedicated systems for collecting unused or expired household medications were reported in 31 out of 35 countries. These systems were most commonly implemented as national programmes (21 countries), with regional (four countries) and local (six countries) initiatives occurring less frequently. However, three countries - Latvia, Montenegro, and North Macedonia - reported having no formal collection system.

**FIGURE 1:**
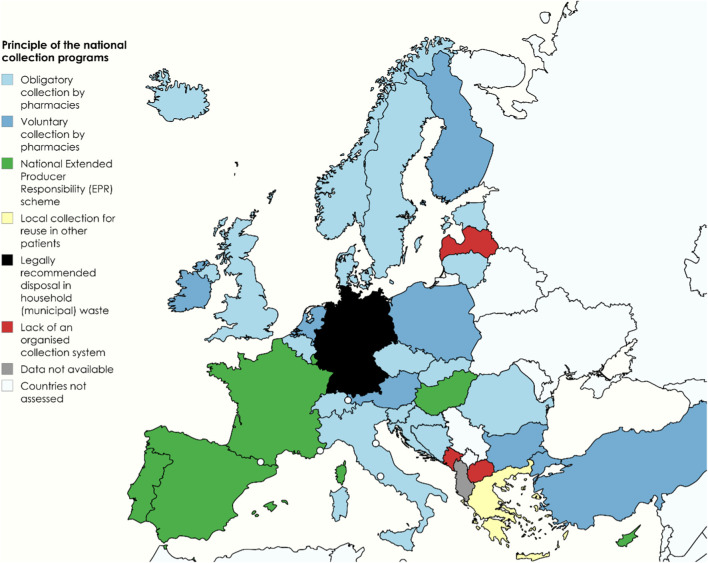
Core mechanisms of national systems for collecting unused or expired household medications. Map created with MapChart (https://www.mapchart.net) under CC BY-SA 4.0.

The core mechanism of these programmes typically involves either mandatory (16 countries) or voluntary (eight countries) collection of unused or expired medications through community pharmacies (Figure 1). In six countries—Cyprus, France, Hungary, Luxembourg, Portugal, and Spain—the collection of this waste is governed by the principles of the polluter-pays principle and the Extended Producer Responsibility scheme. In contrast, Germany reported a legally recommended practice of disposing of medicines in household waste. Notably, in Greece, unused medicines are collected solely through physicians and pharmacists for potential redistribution to individuals who cannot afford their treatment.

Regarding the reuse of collected medications, experts reported that in most countries (24/35) such practices are legally prohibited. Only four countries - Italy, Luxembourg, Spain, and Switzerland - indicated that reuse for humanitarian purposes occurs frequently, while in five others it takes place only occasionally. Netherlands reported that redispensing of medicines is illegal unless conducted within a research setting. A hospital-based pilot programme, launched in 2023, enables the redispensing of selected anti-cancer drugs (Smale et al., 2024a).

Finally, most countries reported having dedicated programmes for the safe disposal of used prefilled syringes, injectors, or similar devices. Among them, 12 operated national programmes, two had regional systems, and nine relied on local or municipal initiatives. An example of such project is Returpen (Returpen TM, 2024), a Danish nationwide take-back scheme for used injection pens (e.g., insulin pens), collecting them so their materials (e.g., plastic, glass) can be recycled. It is funded by a partnership between several pharmaceutical companies and works in close collaboration with pharmacies, healthcare institutions and other stakeholders. According to data provided on its website, up to April 2025, as many as 3 million pens have been collected. Nonetheless, 10 countries reported the absence of any such dedicated programme.

### Practice of disposal of unused drugs

**FIGURE 2:**
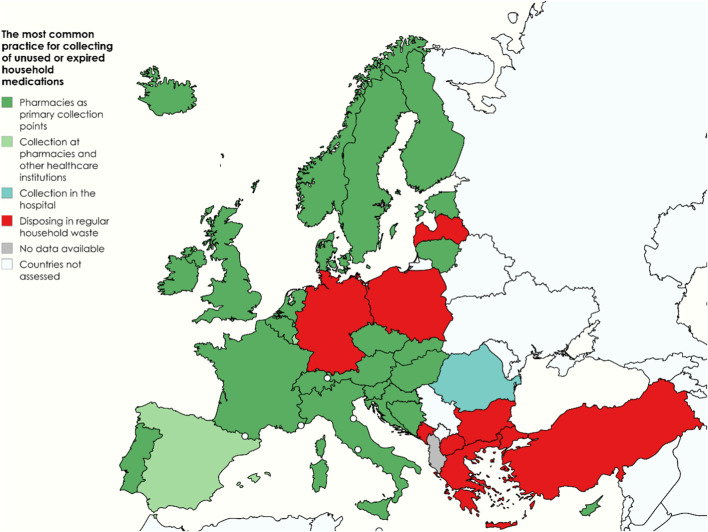
The most common practices for collecting unused or expired household medications reported by the countries studied. Map created with MapChart (https://www.mapchart.net) under CC BY-SA 4.0.

A central focus of the survey was to document the real-world practices employed across participating countries for collecting unused or expired household medications. Community pharmacies were reported as the primary collection point in 25 countries, in two of which (Luxembourg and Spain) they operated alongside other healthcare institutions. In contrast, in Romania, hospitals, via hospital pharmacies, served as the main collection sites (Figure 2).

In contrast, eight countries (Bulgaria, Germany, Greece, Latvia, Montenegro, North Macedonia, Poland, and Türkiye) reported that unused or expired household medications are typically discarded in regular household waste. Evidence from Greece indicates an additional behavioural pattern: a study conducted in rural Crete identified substantial drug stockpiling (an average of 8.5 packs per household) and a very high prevalence of medicine exchange, with 95% of households reporting sharing medications with family or friends (Tsiligianni et al., 2012).

**FIGURE 3:**
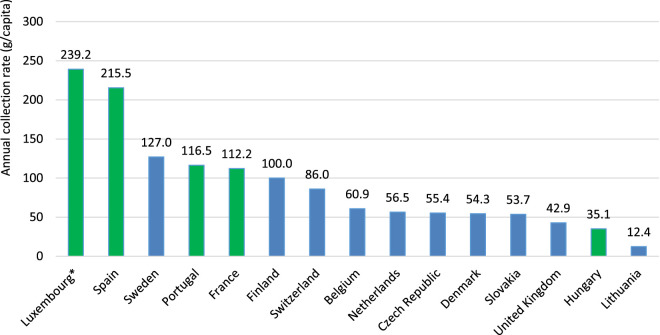
Annual *per capita* collection rates of household pharmaceutical waste across countries (g/capita). Green bars indicate countries with an Extended Producer Responsibility (EPR) system in place. NOTE: *Data include both medicines and syringes; differences between countries with and without EPR schemes were assessed using Fisher’s exact test (P = 0.2399). Despite the lack of statistical significance, the effect size was large (Cramér’s V = 0.564), indicating a strong association.

Figure 3 shows the annual collection rates of household pharmaceutical waste across different countries, ranging from 12.4 g/capita annually for Lithuania to 239.2 g/capita annually in Luxembourg (this number includes collected syringes also). In Germany, since unused medications are officially recommended to be disposed of with household waste, there are no national statistics on the quantities of medicines collected for proper disposal. The estimated volume of discarded pharmaceuticals is approximately 120 g per person per year (Penzel, 2022).

The survey shed some light on barriers to the safe disposal of unused or expired drugs. Limited access to collection points was frequently mentioned, particularly in rural regions. As noted in Scotland: “Rural and dispersed populations in Scotland–In rural Scotland, especially the Highlands, accessing take-back schemes is challenging due to long travel times (often over 2 h). While urban areas (home to ∼70% of the population) face fewer geographic barriers, issues like mobility, stigma, or pharmacy capacity (e.g., full bins) can still limit participation.” Even in countries with established national collection programmes, such as Croatia, certain barriers persist: “Some pharmacies hesitate to accept used sharps or expired drugs due to costs and regulatory burdens associated with handling hazardous waste.” On the other hand, there is also an interesting example of positive reinforcement: Sweden reported a loyalty program which rewards customers when medicines are returned (Organisation for Economic Co-operation and Development, 2022).

Regarding final disposal methods for collected medications, most countries (25) indicated incineration. However, two countries with established collection systems—Croatia and Italy—reported landfilling as their primary method.

### Guidance for patients

The survey explored whether medications distributed in each country typically include information on the proper disposal of unused or expired drugs on the packaging and/or in the patient information leaflet. A positive response was provided by the vast majority (25/35) of countries, most of which reported generic statements in patient leaflets, such as: “Medicines must not be disposed of down the drain or with household waste. Seek advice from a pharmacy on how best to dispose of unused medications. The goal is to protect the environment.” In contrast, nine countries indicated that no such guidance was provided, and not a single country reported the presence of written or pictorial disposal instructions directly on the medication packaging.

The survey also explored whether any national or regional public awareness campaigns promoting the proper disposal of unused or expired medications had been organized within the last 5 years. A total of 22 countries reported such campaigns which varied widely, ranging from small local initiatives to large coordinated national programmes. At the local level, many cities publish their own guidance - for example, on municipal websites in Graz (Austria), Cork (Ireland), and various locations in Estonia, Poland, and Romania - offering practical instructions on medicine storage, disposal, and return options. Moreover, several countries conduct structured, large-scale national activities. For example, in the Netherlands, interventions such as the STOWA–KIWK pharmacy-based pilot, the Week van Ons Water, and the #brengonsterug campaign combine posters, flyers, TV spots, and online messages. Hungary’s Chamber of Pharmacists launched a nationwide initiative titled “Do not throw it in the trash!”. In Finland, the annual Pharmaceutical-free Baltic Sea campaign and National Medication Days have promoted responsible disposal and sustainable medicine use. Belgium developed a national sorting guide during European Waste Reduction Week, while Lithuania, Poland and Portugal have organised national media and educational programmes. Spain operates the well-established SIGRE system, promoting medicine recycling under the motto “Recycling is the best treatment for the planet.” In Slovakia, the State Institute for Drug Control coordinates national campaigns featuring leaflets, videos, and the Drug Amnesty Week. Norway, Sweden, and the United Kingdom run periodic national or multi-regional campaigns through television, pharmacies, and cross-sector collaboration with health boards, water agencies, and environmental organisations. Türkiye linked its public messaging with World Antimicrobial Awareness Week, emphasising proper disposal as a means of reducing environmental contamination. The German Federal Environment Agency (Umweltbundesamt) provides guidance on the safe disposal of household medication waste, emphasizing that, under local regulations, it should be discarded with regular household waste rather than via sewage or toilets. The agency also offers educational materials for pharmacy and medical students on proper disposal practices and broader environmental pharmacy topics.

Only a few countries, however, reported evaluating the effectiveness of their campaigns. In Portugal, Spain and France, where the local EPR operate (these being ValorMed, SIGRE, and Cyclamed, respectively), regular reports showing the scale and impact of their pharmaceutical waste collection are published (Quiroz-Razo and Saturno-Hernández, 2025; VALORMED – Sociedade Gestora de Resíduos de Embalagens e Medicamentos, 2024; Cyclamed, 2025). Italy’s Banco Farmaceutico similarly publishes detailed annual reports on the collection and redistribution of non-expired medicines, which constitute the core of its activities (Gianino et al., 2022). However, in other countries, evaluations are limited or lack quantitative data, making it difficult to assess the true impact of these initiatives. Moreover, many countries show a low level of public knowledge and awareness regarding the proper disposal of unused and expired medications. For example, in some regions, citizens are not fully informed (*“Unfortunately, not all citizens are informed of the regional schedule and do not follow the rules.”*). Similarly, in Germany, outdated online information can lead to improper disposal (*“The website arzneimittelentsorgung.de is no longer financed and therefore information is sometimes outdated, which can lead to improper disposal even when patients wish to do it correctly.”*). Finally, it is noteworthy that 11 countries reported no public awareness campaigns of this kind within the last 5 years.

### Economic aspects

Among the surveyed countries, pharmacies collecting unused or expired household medications are reimbursed for the associated costs in only seven cases (Czech Republic, Denmark, Finland, Italy, Lithuania, Netherlands, and the United Kingdom). The remaining countries reported a lack of such reimbursement mechanisms.

Only a few countries were able to provide quantitative data on the estimated annual costs associated with the collection and disposal of unused or expired household medications, reflecting substantial variability in data availability across national systems (Table 1). Total annual costs were reported to range from around €0.85 million in Hungary to over €3.2 million in the United Kingdom, while *per capita* expenditures spanned from approximately €47 per 1,000 inhabitants in the United Kingdom to €175 per 1,000 inhabitants in Slovakia.

**TABLE 1:** Estimated annual costs of collecting and disposing of unused or expired household medications per country, both total and per 1,000 inhabitants.

Country	Population (million)	Annual cost of collecting and disposing of unused or expired household medications (€)	
		Total	per 1,000 inhabitants
Hungary	9.5	850,000	89.47
Slovakia	5.5	960,000	174.55
Sweden	10.5	1,800,000	171.43
United Kingdom*	69.5	3,266,000	46.99

^*^ Extrapolated based on an incineration cost of £967 per tonne of collected unused drugs for NHS, Scotland in 2024/25, and a total annual collection of 2,968 tonnes across the United Kingdom.

### Household drug waste statistics

Only five countries reported the availability of some statistics on the reasons for household drug waste. In Estonia, unused medicines mostly resulted from leftover doses after completing a course of treatment (71% of respondents). Such medicines were kept because there is no reason or time to dispose of them (49%), or because respondents believed they might be needed in the future (47%) (Rait Faktum & A riko, 2021). In Finland, the waste was primarily driven by limited shelf life after opening (35.5%), improvement of the condition or symptom (24.8%), and unnecessarily large medicine packs (21.5%) (Louhisalmi et al., 2024). In the Netherlands, the most common reasons for returning medications to pharmacies were patient death (22.4%), resolution of the medical condition (19.9%), and medicines past their expiry date (14.6%) (Bekker et al., 2018). In Sweden, a study among pharmacy customers identified expired medications (22%), death of the patient (19%), improved health (18%), and treatment changes (23%) as the main reasons for unused pharmaceuticals. In contrast, in Türkiye the drivers of household accumulation of unused or expired medications differed: they stemmed primarily from intentions to reuse them (41.0%), keep them for emergencies (36.1%), or share with others (17.8%) (Köksoy, 2024). Of note, all these statistics originated from research initiatives rather than public data sources.

Analysis of the top unused or expired medications collected across six countries that provided such data reveals certain patterns in pharmaceutical waste, reflecting both prescribing practices and patient behaviour. Cardiovascular drugs frequently appeared among the most wasted medicines, particularly in the Czech Republic and the Netherlands (ranked first in both countries). Painkillers and anti-inflammatory drugs were also highly represented, with several countries reporting paracetamol and NSAIDs (such as ibuprofen, dexketoprofen, and diclofenac) among the top returned items. Antibiotics (such as amoxicillin with clavulanic acid, ciprofloxacin, and metronidazole), especially in Türkiye and Denmark (ranked first and second, respectively), as well as drugs of ATC (Anatomical Therapeutic Chemical) class A (alimentary tract and metabolism), were also commonly returned.

## Discussion

In this Europe-wide assessment of household medication disposal practices, we provide the first expert-validated overview of how disposal systems operate across diverse national contexts. By integrating legal, organisational, operational, awareness-related, and economic dimensions within a single analytical framework, our findings go beyond the scope of earlier publications. Hence, the study provides a robust basis for future policy and regulatory decision-making on the management of unused and expired household medicines across Europe.

The study highlights substantial cross-country heterogeneity in legislative foundations, organisational infrastructure, and, most importantly, real-world disposal practices for unused household medicines. While most countries have established legal frameworks and pharmacy-based collection systems, their scope and implementation are highly inconsistent, with five lacking specific legislation, three without formal collection mechanisms for medicines, and ten for syringes, injectors and related devices. Even where national programmes exist, collection effectiveness varies markedly, with some populations routinely returning medicines to pharmacies, while in others disposal via household waste or sewage remains the dominant practice. Of note, as many as eight of the 35 countries studied reported discarding unused and expired drugs primarily in household waste, with Germany being the only case where this approach was officially recommended, on the grounds that municipal residual waste in Germany is predominantly incinerated. This variability is reflected in previous local studies, which indicate that over 80% of French and Finnish citizens return unused or expired medicines to pharmacies (Cyclamed, 2025; Louhisalmi et al., 2020), whereas in several Baltic Sea countries and Bulgaria, up to 80% dispose of them via household waste (Mitkova et al., 2023; Käsmann et al., 2024; Mehtonen et al., 2020). Finally, there is substantial diversity in annual collection rates, which vary twenty-fold between Lithuania and Luxembourg. These patterns highlight a persistent gap between policy design and real-world implementation. Thus, our results indicate a clear need for harmonised EU-level standards to ensure more consistent, efficient, and accountable collection and disposal.

Household pharmaceutical waste imposes a substantial economic burden on health systems, municipalities, and governments. Globally, up to half of household medicines are wasted, representing a similar proportion of household medicine spending (Jafarzadeh et al., 2021; Organisation for Economic Co-operation and Development, 2022). In the United States, this proportion is even higher, with two-thirds of sold drugs becoming unused, generating costs of 2.4–5.4 billion USD (Law et al., 2015). In Europe, the economic value of unused medicines accounts for €55 million annually in Denmark (Blenstrup et al., 2024), €81 million annually in Finland (Louhisalmi et al., 2024), €200 million annually in Italy (Romanelli and Lucente, 2022), and up to £800 million annually in the UK (Webb, 2014). Our findings also highlight additional costs connected with collecting and disposing of unused drugs, which were worth millions of euros, despite likely being underestimated due to incomplete reporting.

Analysis of our data shows that countries with an EPR mechanism achieve the highest collection rates due to full national coverage, patient-friendly pharmacy-based collection points, and effective awareness campaigns. Therefore, expanding the EPR scheme across Europe seems advisable as a method to optimise the collection of unused and expired household medicines, while securing stable financing and reducing the economic burden on pharmacies and public budgets.

In most EU countries in general, and all the EPR countries in particular, community pharmacies constitute the primary collection points. Unfortunately, only a minority of surveyed countries reimburse pharmacies for the costs of collecting unused or expired medicines, leaving most systems without dedicated financial support, which limits both efficiency and coverage. Evidence shows that where pharmacies are mandated to accept unused medicines but must absorb the associated costs, collection volumes fall below the average (Jonjić and Vitale, 2014). This funding gap represents a clear policy priority, as highlighted in a recent Pharmaceutical Group of the European Union (PGEU) report calling for adequate reimbursement of pharmacy-led collection schemes (Pharmaceutical Group of the European Union PGEU, 2023).

Our results further underscore an urgent need for systematic data collection and monitoring, as many countries lack reliable statistics on the drivers, volume, and composition of household pharmaceutical waste. Available evidence largely comes from isolated research initiatives rather than routine national reporting, leaving policymakers without a comprehensive evidence base. Standardised monitoring frameworks are needed to allow trend tracking, benchmarking of take-back systems, and more effective policy and resource allocation.

Undoubtedly, the best strategy to consider is the prevention of household pharmaceutical waste at its source. It requires improved prescribing and dispensing practices, including medicines optimisation approaches that ensure appropriate dosing and treatment duration (Paut Kusturica et al., 2022). Analysis indicates that leftover medicines mainly arise from large pack sizes, limited shelf life after opening, as well as treatment changes and symptom resolution. Special attention should be paid in this process to drugs that carry an increased risk of being unused and that have the potential to cause harm. According to the study, waste was concentrated primarily in cardiovascular drugs, antibiotics, NSAIDs, and analgesics. All these categories constitute clear priorities for intervention, with antibiotics being of particular concern due to their contribution to antimicrobial resistance. Once optimal therapy is prescribed, another important goal for interventions is the optimal execution of the treatment by patients. As non-adherence accounts for up to 50% of medication waste, adherence-enhancing strategies therefore represent a complementary opportunity (Jafarzadeh et al., 2021; Organisation for Economic Co-operation and Development, 2022; Makki et al., 2019; Kardas and Agh, 2025).

Of course, even the most effective prevention strategy cannot eliminate medicine waste completely. Hence, widespread knowledge of proper procedures in societies is of crucial importance. Our survey data suggest that low public awareness remains a key driver of improper disposal, largely due to inconsistent, generic, or absent guidance. Although disposal information is often included in patient leaflets, it is typically formulaic and insufficiently visible to influence behaviour, while guidance provided directly on medicine packaging appears to be largely lacking. Public campaigns vary widely in scale and quality, and their effectiveness is rarely evaluated. Several countries report no campaigns at all, while others rely on outdated or inaccessible information. An Estonian survey illustrates this gap, with 77% of respondents preferring to receive disposal guidance from pharmacies, yet only 3% recalling having received such information (Rait Faktum and Ariko, 2021). To address this, several European stakeholder organisations have launched the #medsdisposal initiative (Medsdisposal.eu, 2019). However, much of the content on its website has not been updated since 2020 and remains general, offering limited practical guidance for end users. These limitations highlight that current awareness-raising efforts remain insufficient. Nevertheless, addressing persistent knowledge gaps among the public represents a low-cost but potentially high-impact intervention. Standardised disposal instructions on medication packaging, harmonised disposal symbols at the EU level, and clearer guidance within patient information leaflets could normalise appropriate disposal behaviours. Integration of disposal guidance into the Summary of Product Characteristics and regulatory documentation may further institutionalise environmental responsibility within pharmaceutical lifecycle management.

Another partial remedy for medicine waste could be a rational reutilization and recycling approach. There is evidence that many drugs collected for disposal are potentially reusable—for example, one study showed that returned medicine packages in Italy contained, on average, 68% of the originally dispensed dosage units (Romanelli and Lucente, 2022). Similar conclusions have been reported from an experimental study, suggesting that the controlled reuse of unused oral anticancer drugs can meaningfully reduce waste and improve environmental sustainability (Smale et al., 2024b). However, our findings reveal that approaches to reuse are highly heterogeneous across Europe, with most countries prohibiting reutilization and only a few permitting or piloting controlled redispensing. Management of potentially recyclable material contained in used injection devices mirrors this fragmentation. Only recently has the IHI ENKORE Project begun to address this issue at the European forum, although it remains at an early stage (ENKORE Eco Healthcare, 2022).

Among the limitations of this study, it should be acknowledged that the analysis focused exclusively on the management of household medicinal waste, which represents only one component of the broader issue of pharmaceutical waste. In addition, data availability varied across countries and, in some cases, relied on self-reported estimates, introducing the potential for reporting bias. As the study was based on expert-reported national information, there was also potential for reporting bias arising from subjective interpretation, incomplete knowledge of country-specific practices, or variability in access to official data. Finally, as an expert-based survey, the study could not comprehensively account for behavioural, socioeconomic, and cultural determinants of disposal practices at the level of individual medicine users, except where locally conducted research was available. Nevertheless, as the first Europe-wide study in this field, the survey offers a valuable snapshot of current practices and provides a foundation for future research and corrective policy actions.

Importantly, awareness of the scale and consequences of improper household medicine disposal is gradually increasing across Europe. In this context, European experts involved in this study have endorsed the Brussels Declaration, which calls for a coordinated and responsible approach to the disposal of unused and expired household medicines (Kardas et al., 2025). The Declaration articulates a clear triple objective: the implementation of sustained and coordinated public awareness campaigns; the introduction of harmonised, easily recognisable packaging symbols accompanied by clear disposal instructions; and the development of accessible, regularly updated digital tools to guide citizens to authorised return points and applicable national rules. Together, these measures provide a concrete and actionable framework for translating existing knowledge into practice. Their effective implementation could mark an important step towards closing the gap between policy intent and real-world disposal behaviour across Europe.

## Data Availability

The raw data supporting the conclusions of this article will be made available by the authors, without undue reservation.

## Disposal collaborators

Margaret Bermingham (Pharmaceutical Care Research Group, School of Pharmacy, University College Cork, Cork City, Ireland), Helena Cardon (Pharmaceutical Care Unit, Faculty of Pharmaceutical Sciences, Ghent University, Ghent, Belgium), Tamás István Dóczi (Syreon Research Institute, Budapest, Hungary), Franz Ferrari (Austrian Chamber of Pharmacists, Vienna, Austria), Ralitza Gauthier (pharma24, Geneva, Switzerland), Catherine Goetzinger (National Health Observatory, Luxembourg City, Luxembourg), Anne Gerd Granas (Department of Pharmacy University of Oslo, Oslo, Norway, MedThings, Fredrikstad, Norway), Jonas Grincevičius (Pharmacy and Pharmacology Center, Institute of Biomedical Science, Faculty of Medicine, Vilnius University, Vilnius, Lithuania), Marie-Christine Grouzmann (Office of the Cantonal Physician, Service de la Santé Publique, Canton of Vaud, Switzerland), Katarina Gvozdanović (Teaching Institute of Public Health “Dr Andrija Štampar”, Zagreb, Croatia), Gaye Hafez (Paris-Saclay University, UVSQ, INSERM UMRS 1018, CESP, Gif-sur-Yvette, France, Department of Pharmacology, Faculty of Pharmacy, Altinbas University, Istanbul, Türkiye), Eelko Hak (Department of PharmacoTherapy, Epidemiology and Economics, Groningen Research Institute of Pharmacy, University of Groningen, Groningen, Netherlands), Maria Teresa Herdeiro (Institute of Biomedicine, Department of Medical Sciences, University of Aveiro, Aveiro, Portugal), Freyja Jónsdóttir(Faculty of Pharmaceutical Sciences, University of Iceland, Reykjavík, Iceland, Pharmacy Services, Landspítali University Hospital, Reykjavík, Iceland), Iva Selke Krulichová (Department of Medical Biophysics, Faculty of Medicine in Hradec Králové, Charles University, Prague, Czechia), Francisca Leiva-Fernández (Andalusian Health Service, IBIMA, University of Malaga, Malaga, Spain), Sophie Liabeuf (Pharmacoepidemiology Department, Amiens University Hospital, Amiens, France), Mella Louhisalmi (University of Eastern Finland, Kuopio, Finland), Enrica Menditto (CIRFF – Centre of Pharmacoeconomics and Drug Utilization Research, Department of Pharmacy, University of Naples Federico II, Naples, Italy), Zornitsa Mitkova (Department of Organization and Economy of Pharmacy, Faculty of Pharmacy, Medical University-Sofia, Sofia, Bulgaria), Lydia Niemi (Environmental Research Institute, University of the Highlands and Islands, Inverness, United Kingdom), Lotte Stig Nørgaard (University of Copenhagen, Copenhagen, Denmark), Anna Oleárová (University Hospital Bratislava, Bratislava, Slovakia), Ana Poceva Panovska (Faculty of Pharmacy, Ss. Cyril and Methodius University in Skopje, Skopje, North Macedonia), Guenka Petrova (Department of Organization and Economy of Pharmacy, Faculty of Pharmacy, Medical University-Sofia, Sofia, Bulgaria), Christos Petrou (Department of Health Sciences, University of Nicosia, Nicosia, Cyprus), Sharon Pfleger (Public Health Department, NHS Highland, Inverness and The Robert Gordon University, Aberdeen, United Kingdom), Mitar Popović (Primary Health Care Center, Cetinje, Montenegro), Marie P. Schneider (School of Pharmacy, University of Geneva, Geneva, Switzerland), Matej Stuhec (Department of Pharmacology, Medical Faculty, University of Maribor, Maribor, Slovenia), Tomas Tesar (Department of Organisation and Management of Pharmacy, Faculty of Pharmacy, Comenius University in Bratislava, Bratislava, Slovakia), Ioanna Tsiligianni (Clinic of Social and Family Medicine, Department of Social Medicine, Faculty of Medicine, University of Crete, Rethymno, Greece, Department of Primary Care and Population Health, Medical School, University of Nicosia, Nicosia, Cyprus), Inga Urtane (Riga Stradins University Faculty of Pharmacy, Rīga, Latvia), Bart van den Bemt (Sint Maartenskliniek/Radboudumc, Nijmegen, Netherlands), Johanna Villén (Department of Pharmacy, Faculty of Pharmacy, Uppsala University, Uppsala, Sweden), Vesna Vujić-Aleksić (Institute for Physical Medicine, Rehabilitation and Orthopaedic Surgery “Dr Miroslav Zotović”, Banja Luka, Bosnia and Herzegovina, Department of Pharmacology, Clinical Pharmacology and Toxicology, Faculty of Medicine, University of Banja Luka, Banja Luka, Bosnia and Herzegovina), Clemens Woitaske-Proske (Department of Pharmaceutical Chemistry, Christian-Albrechts-University of Kiel, Kiel, Germany).
