# Supplementary material for: From medicine cabinets to ecosystems: a Europe-wide assessment of household pharmaceutical waste disposal practices
Source: Front Pharmacol. 2026 Apr 28;17:1788038. doi: 10.3389/fphar.2026.1788038 (PMC13161044; doi:10.3389/fphar.2026.1788038)
Supplement: Supplementary file 1 [file DataSheet2.pdf]

Country	Is there any national legislation that specifically addresses the collection of unused or expired household medications?	A short description of the principal national legislation addressing the collection of unused or expired household medications, along with the year of enactment and a link to the official document of this	If organised medication collection is taking place, can patients provide unused or expired drugs for collection anonymously?	Are there any penalties for citizens for the improper disposal of unused or expired medications (e.g., flushing, burning, or landfilling)?	A short description of these penalties, if applicable	Do drugs distributed in your country typically include information about proper disposal of unused or expired drugs on the packaging and/or in the patient information leaflet?	Is there any special system in place for the collection of unused or expired household medications in your country?	What is the core principle of this program? Choose the one you consider most important.	Please provide a short description of this program (how it operates, and its key features) and include a link to the program's official website or provide references to any relevant publication, if available	Is the reuse of collected medications (e.g., redistributing unused, unexpired drugs to those in need) a common practice in your country?	Is there any special collection program in your country for the safe disposal of used prefilled syringes, injectors etc. (e.g., empty injection pens, insulin pens, adrenaline injectors, slimming drug injectors etc.)?	Please provide a short description of this program (how it operates, and its key features) and include a link to the program's official website or provide references to any relevant publication, if available	What is the most common practice for collecting of unused or expired household medications in your country?	What is the final disposal method for collected unused or expired medications? Choose the one(s) you consider most important.	, . . . ,	, . . . ,	, . . . ,	, . . . ,	In the last five years, have any national or regional public awareness campaigns been organized in your country promoting the proper disposal of unused or expired medications?	If yes, please provide examples of these campaigns, including their key messages, as well as a link to its official website, if available.	Have any of these campaigns been evaluated for effectiveness?	If yes, please provide references to any relevant publications or a link, if available.	Who is responsible for covering the costs of collecting of unused or expired household medications in your country?	If pharmacies collect unused or expired household medications, are they reimbursed for the associated costs?	Who is responsible for covering the costs of the final disposal of collected unused or expired medications?	What is the estimated annual volume of unused or expired household medications in your country (in kilograms/tons, if available)? Please provide reporting year.	, . . . ,	, . . . ,	Are national statistics available on the reasons for household drug wasted (e.g., expired drugs, medication non-adherence, etc.)?
Response	Response	Open-Ended Response	Response	Response	Open-Ended Response	Response	Response	Response	Open-Ended Response	Response	Response	Open-Ended Response	Response	Incineration	Landfill	Recycling (i.e. redistributing collected unused not expired drugs to those in need)	Not applicable - unused or expired drugs are not collected	Other (please specify)	Response	Open-Ended Response	Response	Open-Ended Response	Response	Response	Response	Prescription drugs	Over-the-counter (OTC) drugs	All drugs	Response
Albania	National Waste Act or equivalent regulations	This law establishes the rules for the production, placing on the market, import, export, trade, use, pharmacovigilance, advertising, quality control and inspection of activities related to medicinal products for human use in the Republic of Albania,	Yes	No	, . . . ,	No	, . . . ,	, . . . ,	, . . . ,	, . . . ,	, . . . ,	, . . . ,	, . . . ,	, . . . ,	, . . . ,	, . . . ,	, . . . ,	, . . . ,	, . . . ,	, . . . ,	, . . . ,	, . . . ,	, . . . ,	, . . . ,	, . . . ,	, . . . ,	, . . . ,	, . . . ,	, . . . ,

Austria	Specific decrees or laws recognizing unused or expired household medications as hazardous waste and regulating their management	Abfallwirtschaftsgesetz (AWG) 2002	Yes	Yes	Penalty of up to €360 for consumers, if medications is not disposed properly	Yes	Local program(s) (e.g. municipal system)	Voluntary collection by pharmacies: pharmacies may choose to collect unused or expired drugs but are not legally required to do so	Municipalities are obliged to accept medication waste in household quantities free of charge. Often, the local pharmacies are allowed to collect that waste in the name of the municipality, who then collects the waste from the pharmacy.	It is infrequent	Local program(s) (e.g. municipal system)	This depends on the municipality. Some of them also involve pharmacies.	Pharmacies as primary collection points (pharmacy take-back programs)	Incineration	, . . . ,	, . . . ,	, . . . ,	, . . . ,	Yes	This is also organised locally, for example: https://www.graz.at/cms/beitrag/10437139/8114338/Wie_lagere_und_entsorge_ich_Medikamente.html	No	, . . . ,	Local government/municipalities	No	Local government/municipalities	, . . . ,	, . . . ,	, . . . ,	No
Belgium	National Waste Act or equivalent regulations	KB 21/01/2009 Art. 13: https://etaamb.openjustice.be/nl/koninklijk-besluit-van-21-januari-2009_n2009018031.html. This legislation states that pharmacists are legally obliged to take back expired and unused medicines. This applies to both medicines	Yes	Yes	There are no specific penalties for the improper disposal of unused or expired medications, but they can be seen as hazardous waste. Different legislations apply to different parts of the country. In Flanders, the 'Vlaams Materialendecreet'	Yes	National program	Obligatory collection by pharmacies: pharmacies are legally obliged to collect unused or expired drugs from the public	Pharmacists are legally obliged to take back expired and unused medicines returned by patients and to ensure they are disposed of correctly. These are collected in a specific recipient, often referred to as "the yellow bag". The	Not at all, it is forbidden by the law	Local program(s) (e.g. municipal system)	Needles should be collected in a needle container and taken to the municipal recycling centre. A pre-filled syringe with the needle already attached in an unopened packaging may be disposed of in the yellow bag. FAQ oude en vervallen geneesmid	Pharmacies as primary collection points (pharmacy take-back programs)	Incineration	, . . . ,	, . . . ,	, . . . ,	, . . . ,	Yes	A sorting guide is available to raise awareness among patients and inform them about the correct way to return unused and expired medicines. The guide contains information about what the pharmacist will and will not take back and where	No	, . . . ,	Pharmacies	No	Manufacturers	, . . . ,	, . . . ,	Most recent data of 2023: 659.4 tons collected. On average, in the period 2016-2023, about 650 tons of expired and unused medication was collected annually. In 2022, a record was set of 693 tons unused or expired household medication	, . . . ,

Bosnia and Herzegovina	Other (please specify)	Principal documents adressing the collection of unused or expired household medications are Certification standards for community pharmacies (in entity Republic of Srpska, link: https://www.askva.org/fileadmin/Sertifikacija/standardi/Sertifik	Yes	No	, . . . ,	Yes	Other (please specify)	Obligatory collection by pharmacies: pharmacies are legally obliged to collect unused or expired drugs from the public	There is no specific program. However, community pharmacies are required to take back unused or expired household medicines. As set in certification/accreditation standards, community pharmacies must also have standard operating procedure	Not at all, it is forbidden by the law	Lack of such a program	, . . . ,	Pharmacies as primary collection points (pharmacy take-back programs)	, . . . ,	, . . . ,	, . . . ,	, . . . ,	Export to countries that can properly dispose collected household pharmaceutical waste by incineration (e.g. Austria, Italy, Germany), since Bosnia and Herzegovina does not have required technologies.	No	, . . . ,	Not applicable (no campaigns have been organized in the past five years)	, . . . ,	Pharmacies	No	Pharmacies	, . . . ,	, . . . ,	, . . . ,	No
Bulgaria	National Waste Act or equivalent regulations	Law on waste management in force 13.07.2012, last amended 24.09.2024 r https://legislation.apis.bg/doc/559413/0 Regulation for waste management and maintenance and protection of cleanliness on the territory of Sofia Municipality in force	Yes	Other (please specify)	There is a fine of 300 to 1000 BGN (according to Law)	Yes	Regional program(s)	National Extended Producer Responsibility (EPR) scheme: a dedicated organization, appointed by the authorities, to manage the collection of unused or expired drugs	Medicines are in the hazardous waste category. A schedule for collection of hazardous waste, including medicines, is organized at the municipality level. All people can hand them over to the staff when scheduled to be in their residential area.	Not at all, it is forbidden by the law	Regional program(s)	The explained program for waste controlling via municipality companies is applied for all type pharmaceutical products.	Mobile collection centers	, . . . ,	, . . . ,	, . . . ,	, . . . ,	Not available data. (There is no explained in details. For example a private company published that uses autoclaving and microwave treatments, but incineration is also possible)	No	NA	No	NA	Local government/municipalities	No	Local government/municipalities	Not available data	Not available data	Not available data	No

Croatia	Specific decrees or laws recognizing unused or expired household medications as hazardous waste and regulating their management	Regulation on Medical Waste Management (Pravilnik o gospodarenju medicinskim otpadom). Published in the Official Gazette No. 50/2015 and amended by No. 56/2019. Link:https://narodne-novine.nn.hr/clanci/sluzbeni/2015_05_50_9	Yes	Yes	Improper disposal of unused or expired medications is prohibited, however, specific penalties are not explicitly detailed in the available legal texts.	Yes	National program	Obligatory collection by pharmacies: pharmacies are legally obliged to collect unused or expired drugs from the public	The program operates under Croatian regulations (there is no specific web site) that mandate pharmacies to accept unused medicines from households. Also, municipal recycling centers can serve as official sites for collecting pharmaceutical	It is infrequent	National program	Used prefilled syringes, insulin pens, and similar sharps can be safely disposed of primarily through community pharmacies and some municipal recycling centers equipped with special sharps containers. Proper disposal is	Pharmacies as primary collection points (pharmacy take-back programs)	Incineration	Landfill	, . . . ,	, . . . ,	, . . . ,	No	, . . . ,	Not applicable (no campaigns have been organized in the past five years)	, . . . ,	Collaborative funding by public and private entities (e.g. costs split between producers and municipalities; pharmacies and municipalities).	No	Other (please specify)	, . . . ,	, . . . ,	, . . . ,	Not applicable
Cyprus	National Waste Act or equivalent regulations	Household pharmaceutical waste in Cyprus is regulated by the “Waste (Management of Household Pharmaceutical Waste) Regulations, 2021,” which establish a framework for the safe and proper disposal of unused or expired medicines to protect	Yes	Yes	, . . . ,	Yes	National program	National Extended Producer Responsibility (EPR) scheme: a dedicated organization, appointed by the authorities, to manage the collection of unused or expired drugs	https://medicycle.com.cy/	Not at all, it is forbidden by the law	Lack of such a program	, . . . ,	Pharmacies as primary collection points (pharmacy take-back programs)	Incineration	, . . . ,	, . . . ,	, . . . ,	, . . . ,	Yes	, . . . ,	No	https://www.alphanews.live/health/petas-farmaka-sta-skoupidia-aftoi-einai-oi-kindynoi-gia-ygeia-kai-perivallon/ https://www.unic.ac.cy/el/diadiktyaki-syzitisi-diacheirisi-apovliton-farmakeytikon-proionton-oikiakis-proeleysis/	Manufacturers under Extended Producer Responsibility (EPR) scheme	No	Manufacturers	, . . . ,	, . . . ,	, . . . ,	, . . . ,

Czech Republic	National Waste Act or equivalent regulations	In accordance with Act No. 378/2007 Coll. (Act on Pharmaeuticals), pharmacies are obliged to accept waste pharmaceuticals from natural persons free of charge. Unused household medicines are collected from	Other (please specify)	Not applicable	, . . . ,	, . . . ,	National program	Obligatory collection by pharmacies: pharmacies are legally obliged to collect unused or expired drugs from the public	Pharmacies are obliged to accept waste pharmaceuticals from the public. The waste medicines are collected from pharmacies by authorized persons and transported to incinerators. The pharmacies report quarterly the	Not at all, it is forbidden by the law	Other (please specify)	Pharmacies usually accept empty injectors or unused prefilled syringes. They should also be accepted for disposal by the healthcare facility whose doctor prescribed the treatment for the patient.	Pharmacies as primary collection points (pharmacy take-back programs)	Incineration	, . . . ,	, . . . ,	, . . . ,	, . . . ,	, . . . ,	, . . . ,	, . . . ,	, . . . ,	National government	Yes	Government	, . . . ,	, . . . ,	, . . . ,	, . . . ,
Denmark	Specific decrees or laws recognizing unused or expired household medications as hazardous waste and regulating their management	The Danish Medicines Act (https://www.retsinformation.dk/eli/lta/2023/339) contains provisions regarding the disposal of medicines to ensure they are handled safely and do not pose a risk to people or the environment. According to the law, medicines	Yes	No	, . . . ,	Yes	Other (please specify)	Obligatory collection by pharmacies: pharmacies are legally obliged to collect unused or expired drugs from the public	Information about the proper disposal of medications is often provided to the public through various channels, including pharmacy staff, public health campaigns, and official websites. Key Features Accessibility: Any pharmacy in Denmark	Not at all, it is forbidden by the law	National program	https://www.novonordisk.com/sustainable-business/zero-environmental-impact/recycling-used-devices.	Pharmacies as primary collection points (pharmacy take-back programs)	, . . . ,	, . . . ,	Recycling (i.e. redistributing collected unused not expired drugs to those in need)	, . . . ,	, . . . ,	Yes	together with a collegue I have run a study in this area a few years ago. Data were collected in 39 pharmacies. Please contact me on lotte.norgaard@sund.ku.dk - and I will send you the results from the study	No	, . . . ,	Manufacturers under Extended Producer Responsibility (EPR) scheme	Yes	Manufacturers	, . . . ,	, . . . ,	300 tons from pharmacies (2009). How large a fraction of the total amount is disposed of through household waste and toilets is unknown (Ministeriet for Sundhed og Forebyggelse 2009).	Other (please specify)

Estonia	Specific decrees or laws recognizing unused or expired household medications as hazardous waste and regulating their management	Medicinal Products Act, Subchapter 5 Storage and Transport of Medicinal Products and Handling of Medicinal Products Withdrawn from Market, https://www.riigiteataja.ee/en/eli/ee/516052016002/consolide/current	yes	no	, . . . ,	yes	National program	Obligatory collection by pharmacies: pharmacies are legally obliged to collect unused or expired drugs from the public	see ORIGINAL	Not at all, it is forbidden by the law	Other (please specify)	see ORIGINAL	Pharmacies as primary collection points (pharmacy take-back programs)	Incineration	, . . . ,	, . . . ,	, . . . ,	, . . . ,	Yes	Municipality home pages provide information about disposal of medicines, no direct campains available	No	, . . . ,	Pharmacies	no	Pharmacies	, . . . ,	, . . . ,	, . . . ,	Other (please specify)
Finland	National Waste Act or equivalent regulations	The national Waste Act mandates that hazardous waste must be collected separately from other waste, and the collection of hazardous waste from households is the responsibility of municipalities (Waste Act 646/2011, first	Other (please specify)	No	Not applicable	Yes	National program	Voluntary collection by pharmacies: pharmacies may choose to collect unused or expired drugs but are not legally required to do so	Municipalities have agreements with pharmacies to collect household pharmaceutical waste. Although voluntary, in practice, pharmaceutical waste can be returned to all Finnish pharmacies free of charge. Only a few municipalities allow returns to	Not at all, it is forbidden by the law	Other (please specify)	Generally they were collected in the same collection program as pharmaceutical waste. But in some areas, waste management facilities may direct empty needles to mixed waste.	Pharmacies as primary collection points (pharmacy take-back programs)	Incineration	, . . . ,	, . . . ,	, . . . ,	, . . . ,	Yes	National Pharmaceutical-free Baltic Sea campaign annually 2018-2022 Encouraged returning pharmaceutical waste to the pharmacy, website not available. National Medication Days, themed around sustainable and responsible use of medicines,	No	, . . . ,	Local government/municipalities	Other (please specify)	Local government/municipalities	, . . . ,	, . . . ,	About 5.6 million medicine pack are returned to pharmacies annually, 2024	Yes

France	Other (please specify)	Pharmacies are required to collect Unused Medicines (UHMs), including, where applicable, their packaging, free of charge (Articles R. 4211-23 and following of the French Public Health Code). These medicines are destroyed	Yes	No	, . . . ,	No	National program	Obligatory collection by pharmacies: pharmacies are legally obliged to collect unused or expired drugs from the public	The system for managing Unused Medicines (UHMs) was established in 1993, following a European directive. It was created to handle the collection of unused and expired medicines from individuals. The UHM system has been officially approved	Not at all, it is forbidden by the law	Lack of such a program	https://www.dastri.fr/ : Specific for insulin pens	Pharmacies as primary collection points (pharmacy take-back programs)	Incineration	, . . . ,	, . . . ,	, . . . ,	, . . . ,	No	, . . . ,	Not applicable (no campaigns have been organized in the past five years)	, . . . ,	National government	No	Government	, . . . ,	, . . . ,	3 Mrd	No
Germany	Municipal rules for waste handling	There is no common national legislation, but municipal rules to dispose unused medication via household waste, mobile waste collection points, recycling centers or in two communities via pharmacies. Correct disposal can be found at	Other (please specify)	No	, . . . ,	Yes	Local program(s) (e.g. municipal system)	Legally recommended disposal of unused or expired drugs in household (municipal) waste: authorities recommend disposing of unused or expired drugs in regular trash bins	No specific collection system, but household waste is burned before it is deposited because unused medicine shall be disposed and rendered harmless through this way. If household wate is not burned, it is collected via mobile waste collection or	Other (please specify)	Local program(s) (e.g. municipal system)	Disposal via household waste.	Disposing of them in regular household waste (standard waste collection with household rubbish)	Incineration	, . . . ,	, . . . ,	, . . . ,	, . . . ,	Other (please specify)	, . . . ,	No	, . . . ,	Local government/municipalities	No	Local government/municipalities	, . . . ,	, . . . ,	about 30 % of all purchased drugs. In 2011, about 1.4 billion packages were dispensed.	No
Greece	No specific legislation in place	, . . . ,	Not applicable	No	, . . . ,	No	Other (please specify)	Other (please specify)	, . . . ,	It is infrequent	National program	Collection in practices and then transport to regional sites as regards PC	Disposing of them in regular household waste (standard waste collection with household rubbish)	, . . . ,	, . . . ,	, . . . ,	Not applicable - unused or expired drugs are not collected	, . . . ,	Yes	, . . . ,	No	, . . . ,	Not applicable	Not applicable	Not applicable (no collection takes place)	, . . . ,	, . . . ,	, . . . ,	Not applicable

Hungary	Specific decrees or laws recognizing unused or expired household medications as hazardous waste and regulating their management	12/2017 (VI. 12) EMMI Decree on waste management activities related to waste generated by healthcare providers (12/2017. (VI. 12.) EMMI rendelet az egészségügyi szolgáltatónál képződő hulladékkal kapcsolatos	Yes	Yes	Administrative fine can be applied to individuals, this is determined on the basis of Act CLI of 2012. It's not widely applied in practice, because it is very difficult to establish a violation in the case of private individuals.	Yes	National program	National Extended Producer Responsibility (EPR) scheme: a dedicated organization, appointed by the authorities, to manage the collection of unused or expired drugs	Recyclomed Kft. has been the sole coordinator of collection, with nationwide coverage. The company designed and finalized the green collection boxes placed in pharmacies and drugstores, where expired and unused medicines	Not at all, it is forbidden by the law	National program	Since 12 February 2019, all outpatient and inpatient healthcare providers must set up special collection points where the public can safely drop off used needles, syringes and infusion sets, as required by Decrees 11/2017 and 12/2017 of the	Pharmacies as primary collection points (pharmacy take-back programs)	Incineration	, . . . ,	, . . . ,	, . . . ,	, . . . ,	Yes	Don't throw it in the trash! – What should we do with expired medicines? (Hungarian Chamber of Pharmacists, https://www.facebook.com/magyargyogyszereszikamara/posts/ne-dobja-a-kuk%C3%A1ba-mit-tegy%C3%BCnk-a-lej%C3%A1rt-	Other (please specify)	, . . . ,	Manufacturers under Extended Producer Responsibility (EPR) scheme	Not applicable	Manufacturers	56.6% (2023 study on 113,3 kg analysed waste)	41.1% (2023 study on 113,3 kg analysed waste)	338.2 tonnes (2023)	No
Iceland	National Waste Act or equivalent regulations	The Medicinal Products Act, the Icelandic Medicines Agency oversees the regulation of medicinal products, including aspects related to their disposal.	Yes	No	, . . . ,	Other (please specify)	National program	Obligatory collection by pharmacies: pharmacies are legally obliged to collect unused or expired drugs from the public	Pharmacies are obligated to collect unused medication by law.	Not at all, it is forbidden by the law	National program	Iceland has established systems for the safe disposal of used prefilled syringes, injection pens, and similar medical devices. Pharmacies across the country accept these items to ensure they are disposed of properly and do not pose a risk	Pharmacies as primary collection points (pharmacy take-back programs)	Incineration	, . . . ,	, . . . ,	, . . . ,	, . . . ,	No	there was a campaign 7 years ago.https://www.lyfjastofnun.is/frettir/atakid-bar-arangur/	Yes	https://www.lyfjastofnun.is/frettir/atakid-bar-arangur/	Pharmacies	No	Other (please specify)	, . . . ,	, . . . ,	, . . . ,	No

Ireland	National Waste Act or equivalent regulations	Waste Management Act 1996, as amended. https://www.irishstatutebook.ie/eli/1996/act/10/enacted/en/html	Yes	Other (please specify)	, . . . ,	Yes	Regional program(s)	Voluntary collection by pharmacies: pharmacies may choose to collect unused or expired drugs but are not legally required to do so	Dispose of Unused Medicines Properly (DUMP) campaign is organised by the Health Service Executice in conjunction with community pharmacists in the Cork/Kerry region since 2007. DUMP encourages the public to return	Not at all, it is forbidden by the law	Other (please specify)	https://www.tcp.ie/pages/sharps-waste-management-services; https://hibernianhealth.com/patient-services/	Pharmacies as primary collection points (pharmacy take-back programs)	Incineration	, . . . ,	, . . . ,	, . . . ,	, . . . ,	Other (please specify)	https://www.corkcity.ie/en/council-services/services/environment/environmental-awareness/dump/ AND https://www.nsrf.ie/dispose-of-unused-medicines-properly-dump-campaign-march-14th-to-april-22nd/ AND https://www.echolive.ie/corkne	Other (please specify)	, . . . ,	Pharmacies	No	Pharmacies	, . . . ,	, . . . ,	4.5 tonnes (2023) Cork/Kerry region only	No
Italy	National Waste Act or equivalent regulations	Legislative Decree 24 April 2006, n. 219 address the collection of unused or expired household medications in Italy. DPR 254/2003 regulates their management. Italian Regulatory Agency (Det. 821/2018) updates criteria for the disposal of pharmace	Yes	No	Law No. 3/2018 (the so-called Lorenzin Law), in case of possession in the pharmacy of broken, expired or imperfect drugs there is a fine of € 1,500.00 to € 3,000.00 intended for the pharmacist owner, if it can be concretely excluded that the (expired)	Yes	Local program(s) (e.g. municipal system)	Obligatory collection by pharmacies: pharmacies are legally obliged to collect unused or expired drugs from the public	When the pharmaceutical products expired, the households can waste them in the storage box (generally outside community pharmacies) identified by Assinde. Each package is individually registered	It is frequent	Local program(s) (e.g. municipal system)	“Banco Farmaceutico” program includes laws and regional initiatives for the recovery, reuse, and donation of medications. https://www.bollettinosifo.it/archivio/2688/articoli/27495/#:~:text=La%20possibilit%C3%A0%20di%20riutilizzare%20i,219%20del	Pharmacies as primary collection points (pharmacy take-back programs)	, . . . ,	Landfill	Recycling (i.e. redistributing collected unused not expired drugs to those in need)	, . . . ,	, . . . ,	Yes	https://www.bancofarmaceutico.org/cosa-facciamo/recupero-farmaci-validi ; https://www.ordinefarmacistinapoli.it/news/1097-un-farmaco-per-tutti ; https://www.novonordisk.it/sustainable-business/ReMed.html	Yes	https://www.bancofarmaceutico.org/cosa-facciamo/recupero-farmaci-validi/rapporto-annuale	Manufacturers under Extended Producer Responsibility (EPR) scheme	Other (please specify)	Manufacturers	, . . . ,	, . . . ,	, . . . ,	Not applicable
Latvia	National Waste Act or equivalent regulations	Voluntary collection by pharmacies	Yes	no	, . . . ,	No	Lack of such a program	, . . . ,	, . . . ,	Not at all, it is forbidden by the law	Lack of such a program	, . . . ,	Disposing of them in regular household waste (standard waste collection with household rubbish)	, . . . ,	, . . . ,	Recycling (i.e. redistributing collected unused not expired drugs to those in need)	, . . . ,	, . . . ,	No	, . . . ,	No	, . . . ,	Pharmacies	No	Pharmacies	approx. 13 tons (in 2024)	approx. 7 tons (in 2024)	approx. 20 tons (in 2024)	No

Lithuania	National Waste Act or equivalent regulations	Republic of Lithuania Law on Pharmacy, Enactment date:Jun 22, 2006, https://www.e-tar.lt/portal/en/legalAct/TAR.FF33B3BF23DD/asr	Yes	Yes	Environmental pollution with less than one hundred cubic decimeters of hazardous waste incurs a fine of sixty to one hundred and forty euros.	Yes	National program	Obligatory collection by pharmacies: pharmacies are legally obliged to collect unused or expired drugs from the public	Community pharmacies must accept pharmaceutical waste from the population free of charge. https://sam.lrv.lt/lt/veiklos-sritys/farmacine-ir-kita-su-tuo-susijusi-veikla/informacija-vaistinems-ir-farmacijos-specialistams/informacija-	Not at all, it is forbidden by the law	Lack of such a program	, . . . ,	Pharmacies as primary collection points (pharmacy take-back programs)	Incineration	, . . . ,	, . . . ,	, . . . ,	, . . . ,	Yes	The National Health Insurance Fund under the Ministry of Health published an article about this problem. https://ligoniukasa.lrv.lt/lt/naujienos/milijonai-ismestu-euru-ka-rodo-farmaciniu-atlieku-statistika/	No	, . . . ,	National government	Yes	Government	, . . . ,	, . . . ,	2014 y - 18,576 tons, 2015 y - 42,205 tons, 2016 y - 62,028 tons, 2017 y - 84,488 tons, 2018 y - 148,509 tons, 2019 m. 157,029 tons, 2020 y - 182,180 tons, This is collected from pharmacies only - year 2017 - 14,3 tons, year 2018 - 19,4, year 2019 - 21,3, year 2020 - 28,5 tons; year	No
Luxembourg	No specific legislation in place	, . . . ,	No	No	, . . . ,	No	National program	National Extended Producer Responsibility (EPR) scheme: a dedicated organization, appointed by the authorities, to manage the collection of unused or expired drugs	, . . . ,	Other (please specify)	National program	, . . . ,	Other (please specify)	, . . . ,	Landfill	, . . . ,	, . . . ,	, . . . ,	No	THere was a campaign in 2015. for which no effectiveness evaluation was conducted	, . . . ,	, . . . ,	National government	No	Government	, . . . ,	, . . . ,	around 380 tonnes of pharmaceuticals	No
Montenegro	No specific legislation in place	, . . . ,	Not applicable	Not applicable	, . . . ,	Yes	Lack of such a program	Other (please specify)	, . . . ,	It is infrequent	Lack of such a program	, . . . ,	Disposing of them in regular household waste (standard waste collection with household rubbish)	, . . . ,	, . . . ,	, . . . ,	Not applicable - unused or expired drugs are not collected	, . . . ,	No	, . . . ,	Not applicable (no campaigns have been organized in the past five years)	, . . . ,	Not applicable	Not applicable	Not applicable (no collection takes place)	, . . . ,	, . . . ,	, . . . ,	Not applicable

Netherlands	National Waste Act or equivalent regulations	, . . . ,	Yes	Yes	There are penalties for improperly disposing of waste, this will generally be 95 euros.	No	National program	Voluntary collection by pharmacies: pharmacies may choose to collect unused or expired drugs but are not legally required to do so	Municipalities are responsible for the collecting and processing of small chemical waste, which includes medications. Residents can bring their small chemical waste to municipal recycling centers. To provide collection points closer to residents'	Not at all, it is forbidden by the law	Local program(s) (e.g. municipal system)	This is organized in the same way as collection for medications is. Municipalities are responsible but pharmacies aid by functioning as collection points. This waste is also considered as small chemical waste. The one different is	Pharmacies as primary collection points (pharmacy take-back programs)	Incineration	, . . . ,	, . . . ,	, . . . ,	, . . . ,	Yes	STOWA-KIWK: use of flyers and stickers during an intervention period of 3 weeks in 6 pharmacies.<br><br>https://www.stowa.nl/publicaties/pilotinterventie-inleveren-ongebruikte-medicijnen-kiwk<br>Week van Ons Water: yearly	Yes	https://www.stowa.nl/publicaties/pilotinterventie-inleveren-ongebruikte-medicijnen-kiwk	Local government/municipalities	Other (please specify)	Local government/municipalities	300.000.000 DDDs/year	, . . . ,	, . . . ,	Yes
North Macedonia	No specific legislation in place	Law on Waste Management (2004) outlines the principles and objectives for waste management, including the development of strategies, plans, and programs. It defines the rights and obligations of individuals and legal entities	Not applicable	Not applicable	, . . . ,	Yes	Lack of such a program	, . . . ,	, . . . ,	Not applicable (collection does not take place)	Lack of such a program	, . . . ,	Disposing of them in regular household waste (standard waste collection with household rubbish)	Incineration	, . . . ,	, . . . ,	, . . . ,	, . . . ,	No	, . . . ,	Not applicable (no campaigns have been organized in the past five years)	, . . . ,	Not applicable	No	Not applicable (no collection takes place)	not applicable	not applicable	not applicable	No

Norway	National Waste Act or equivalent regulations	https://www.helsenorge.no/medisiner/medisiner-holdbarhet-og-oppbevaring/#kvar-kastar-eg-gamle-eller-ubrukte-medisinar	Yes	No	, . . . ,	Yes	National program	Obligatory collection by pharmacies: pharmacies are legally obliged to collect unused or expired drugs from the public	The digital «one way in» Helsenorge.no states that all unused medicines must be returned to any Pharmacy, and not thriwn in the bin.	Not at all, it is forbidden by the law	National program	Same as above. To be returned to a Pharmacy. Drug addicts may also return syringrs and needles to municipality waste return facilities.	Pharmacies as primary collection points (pharmacy take-back programs)	Incineration	, . . . ,	, . . . ,	, . . . ,	, . . . ,	Yes	National television awareness every few years. Posters in the pharmacies. https://www.nrk.no/ostfold/farmasoyt-oppfordrar-til-a-ikkje-bruke-medisinar-som-har-gatt-ut-pa-dato-1.16863586	Other (please specify)	, . . . ,	Other (please specify)	No	Distributors/Wholesalers	, . . . ,	, . . . ,	, . . . ,	No
Poland	National Waste Act or equivalent regulations	, . . . ,	Yes	No	, . . . ,	Yes	Other (please specify)	Voluntary collection by pharmacies: pharmacies may choose to collect unused or expired drugs but are not legally required to do so	local & temporal	Not at all, it is forbidden by the law	Lack of such a program	, . . . ,	Disposing of them in regular household waste (standard waste collection with household rubbish)	Incineration	, . . . ,	, . . . ,	, . . . ,	, . . . ,	Yes	promotion of pharmacy-based collection; https://lekidoapteki.pl/	No	, . . . ,	Pharmacies	No	Pharmacies	, . . . ,	, . . . ,	, . . . ,	No
Portugal	National Waste Act or equivalent regulations	https://diariodarepublica.pt/dr/detalhe/decreto-lei/48-2015-66953313	Yes	No	, . . . ,	Yes	National program	Voluntary collection by pharmacies: pharmacies may choose to collect unused or expired drugs but are not legally required to do so	https://diariodarepublica.pt/dr/detalhe/despacho/9592-2015-70083894	Not at all, it is forbidden by the law	National program	https://diariodarepublica.pt/dr/detalhe/portaria/301-a-2016-105283970	Pharmacies as primary collection points (pharmacy take-back programs)	Incineration	, . . . ,	, . . . ,	, . . . ,	, . . . ,	Yes	https://valormed.pt/campanha/campanha-2024-por-um-futuro-feito-de-vida-cuide-hoje-do-ambiente/	Yes	https://valormed.pt/quem-somos/relatorios-e-indicadores/	National government	No	, . . . ,	, . . . ,	, . . . ,	, . . . ,	, . . . ,

Romania	Specific decrees or laws recognizing unused or expired household medications as hazardous waste and regulating their management	https://lege5.ro/gratuit/ge2tanbwgy4ts/instructiunea-nr-6226-2024-privind-modalitatea-de-gestionare-a-deseurilor-de-medicamente-neutilizate-si-sau-expirate-provenite-de-la-populatie. Hospitals are responsible for collecting	Yes	Not applicable	, . . . ,	Yes	National program	Other (please specify)	, . . . ,	Not at all, it is forbidden by the law	National program	pharmacies collecting syringes. all biological wastes are collected in the units where are used by authorised firms for biologicl wate distruction	Other (please specify)	Incineration	, . . . ,	, . . . ,	, . . . ,	, . . . ,	Yes	various media informative materials with the general theme: What do you do with expired medicines ?	Other (please specify)	, . . . ,	Healthcare system	, . . . ,	Healthcare system	, . . . ,	, . . . ,	, . . . ,	No
Slovakia	National Waste Act or equivalent regulations	The methodological instruction determines the correct handling and collection of hazardous waste - medicines not consumed by individuals in the premises of a public pharmacy. Legislative regulations: Act No. 79/2015 Coll. on	Yes	No	Under Slovak law, unused or expired medicines are hazardous waste and must be returned to pharmacies, which accept them free of charge. Improper disposal is prohibited and may be fined up to 1,500 EUR, but enforcement against individuals	Yes	National program	Obligatory collection by pharmacies: pharmacies are legally obliged to collect unused or expired drugs from the public	Management of waste from medicines not consumed by individuals in a public pharmacy • A public pharmacy must have a designated area in accordance with the Decree of the Ministry of Health of the Slovak Republic No. 129/2012	Not at all, it is forbidden by the law	National program	Each bag must be marked with a label with the waste catalog number 20 01 31 and the identification data of the establishment (name and address) upon collection. • Unused medicines will be collected from citizens by the responsibl	Pharmacies as primary collection points (pharmacy take-back programs)	Incineration	, . . . ,	, . . . ,	, . . . ,	, . . . ,	Yes	https://www.sukl.sk/hlavna-stranka/english-version?page_id=256	Yes	https://www.sukl.sk/hlavna-stranka/english-version?page_id=256	National government	No	Government	, . . . ,	, . . . ,	291 tonnes (2nd halfyear 2023-2024) https://www.sukl.sk/hlavna-stranka/slovenska-verzia/media/tlacove-spravy/v-roku-2024-sa-vyzbieralo-291-ton-liekoveho-odpadu.-jarny-turnus-zberu-zacina-vo-februari?page_id=6463	No

Slovenia	No specific legislation in place	NO specific legislation, only recommendationy from pharmacists to take medication to pharmacy	No	No	/	No	National program	Obligatory collection by pharmacies: pharmacies are legally obliged to collect unused or expired drugs from the public	Pharmacists collect these medications and everybody could take them to the pharmacy. There are no any obligations by users, except helathcare systems (e.g. hospitals who should collect them via pharmacy and transport to the final	Not at all, it is forbidden by the law	Lack of such a program	/	Pharmacies as primary collection points (pharmacy take-back programs)	Incineration	, . . . ,	, . . . ,	, . . . ,	, . . . ,	No	/	No	/	Pharmacies	No	Distributors/Wholesalers	, . . . ,	, . . . ,	, . . . ,	No
Spain	National Waste Act or equivalent regulations	Waste medicines are subject to a specific separate collection system, using designated containers (called SIGRE Points) located in participating pharmacies. Year: 2001 https://www.miteco.gob.es/es/calidad-y-evaluacion-ambiental/temas/pre	Yes	No	, . . . ,	Yes	National program	National Extended Producer Responsibility (EPR) scheme: a dedicated organization, appointed by the authorities, to manage the collection of unused or expired drugs	SIGRE is a non-profit organization responsible for ensuring the proper environmental management of medication packaging and waste generated in homes. It is supervised by regional governments. Year 2001. https://sigre.es/conocenos/#que-es-sigre	It is frequent	Local program(s) (e.g. municipal system)	Each hospital or primary care institution organizes recycling, based on clean points, recycling of sharps and biological risk in special containers, and SIGRE points. Press release: https://web.sas.junta-andalucia.es/servicioandaluzdesalud/todas-	Collection at pharmacies AND other healthcare institutions (e.g., hospitals, healthcare centers, clinics etc.)	, . . . ,	, . . . ,	Recycling (i.e. redistributing collected unused not expired drugs to those in need)	, . . . ,	, . . . ,	Yes	Recycling is the best treatment for the planet: https://sigre.es/post/reciclar-es-el-mejor-tratamiento-para-el-planeta	No	, . . . ,	Collaborative funding by public and private entities (e.g. costs split between producers and municipalities; pharmacies and municipalities).	No	Other (please specify)	Data not available	Data not available	Data not available	No

Sweden	Obligations established under Extended Producer Responsibility (EPR) scheme	Pharmaceutical waste is governed by three key legislations: the Environmental Code (SFS 1998:808), the Waste Ordinance (SFS 2011:927), and the Ordinance on Producer Responsibility for Pharmaceuticals (SFS 2009:1031). Pharmacie	Yes	No	, . . . ,	Other (please specify)	National program	Obligatory collection by pharmacies: pharmacies are legally obliged to collect unused or expired drugs from the public	In Sweden, a free take-back system is in place for household pharmaceutical waste. Households are responsible for returning leftover medicines to pharmacies, typically using transparent bags to facilitate visual inspection. Pharmacy staff	Not at all, it is forbidden by the law	Local program(s) (e.g. municipal system)	It is the municipality's task to take care of used prefilled syringes, injectors etc. Most municipalities have agreements with pharmacies so that you can pick up and leave needle containers there.	Pharmacies as primary collection points (pharmacy take-back programs)	Incineration	, . . . ,	, . . . ,	, . . . ,	, . . . ,	Yes	All pharmacies provide information on returning unused medicines, though the level of detail varies. Pharmacy chains and regions also run campaigns, such as brochures or videos in healthcare settings. These may target general returns or	No	, . . . ,	Pharmacies	Other (please specify)	Pharmacies	, . . . ,	, . . . ,	1340 tonnes (2024)	Other (please specify)
Switzerland	Other (please specify)	In Switzerland, there is no specific national legislation dedicated exclusively to the collection of unused or expired household medications. However, the management of such waste is governed under broader legislation concerning special waste. The	Yes	Yes	LPE, art. 60 and 61 - https://www.fedlex.admin.ch/eli/cc/1984/1122_1122_1122/fr - There are financial and criminal penalty	No	Other (please specify)	Obligatory collection by pharmacies: pharmacies are legally obliged to collect unused or expired drugs from the public	This is the example of the program of the canton of Geneva: https://www.ge.ch/document/8224/telecharger. It explains that medicines returned by patients and cytostatics must be collected at the pharmacy in defined boxes. It also provides a contact	Other (please specify)	Regional program(s)	Regional and local programmes often linked to pharmacies, certain waste disposal centres or special household waste collection points. Patients are provided secure boxes in which to dispose of sharp objects such as needles and	Pharmacies as primary collection points (pharmacy take-back programs)	Incineration	, . . . ,	, . . . ,	, . . . ,	, . . . ,	Other (please specify)	, . . . ,	Not applicable (no campaigns have been organized in the past five years)	, . . . ,	Local government/municipalities	No	Local government/municipalities	, . . . ,	, . . . ,	43 tonnes in Geneva in 2021, 500'000 inhabitants, which makes 100g/inhabitant.	No

Turkey	Other (please specify)	Environmental Law and Waste Management Regulations: https://www.mevzuat.gov.tr/MevzuatMetin/1.5.2872.pdf The management of waste in Turkey is primarily governed by the Environmental Law (Law No. 2872) enacted in 1983. This law, along with	Not applicable	No	, . . . ,	Other (please specify)	Other (please specify)	Voluntary collection by pharmacies: pharmacies may choose to collect unused or expired drugs but are not legally required to do so	Pharmabotanica is a licensed pharmaceutical cooperative established in Ankara, which is authorised by the Ministry of Environment and Urbanisation under the Environmental Permit and License. Pharmacies can register through	Not at all, it is forbidden by the law	Other (please specify)	Under the Climate Change and Zero Waste Directorate, Bakırköy/Istanbul Municipality has initiated programs to collect expired or unused medications from households. The municipality has placed indoor medication collection boxes in	Disposing of them in regular household waste (standard waste collection with household rubbish)	Incineration	, . . . ,	, . . . ,	, . . . ,	, . . . ,	Yes	World Antimicrobial Awareness Week, by the Turkish Society for Clinical Microbiology and Infectious Diseases (EKMUD), focused on antimicrobial resistance and the importance of medication use and disposal to prevent environmental contamina	No	, . . . ,	Pharmacies	No	Pharmacies	no comprehensive national data	data not available	Estimated total boxes: ~399 million boxes	Other (please specify)
United Kingdom	No specific legislation in place	no legislation for household medicines only healthcare establishment medicines	yes	no	, . . . ,	Yes	Regional program(s)	Obligatory collection by pharmacies: pharmacies are legally obliged to collect unused or expired drugs from the public	NHS boards have to provide a service to enable patients to return meds to their community pharmacy. The waste contractor delivers and collects collection boxes which are taken for incineration. https://www.cps.scot/local-	Not at all, it is forbidden by the law	Local program(s) (e.g. municipal system)	see ORIGINAL	Pharmacies as primary collection points (pharmacy take-back programs)	Incineration	, . . . ,	, . . . ,	, . . . ,	, . . . ,	Yes	see ORIGINAL	no	, . . . ,	Healthcare system	Yes	Healthcare system	, . . . ,	, . . . ,	all meds returned to community pharmacy only 365 tonnes	No
