# Supplementary material for: From medicine cabinets to ecosystems: a Europe-wide assessment of household pharmaceutical waste disposal practices
Source: Front Pharmacol. 2026 Apr 28;17:1788038. doi: 10.3389/fphar.2026.1788038 (PMC13161044; doi:10.3389/fphar.2026.1788038)
Supplement: Supplementary file 2 [file DataSheet1.pdf]

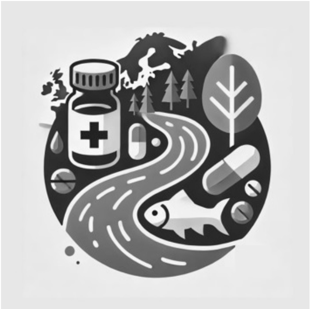

National Expert Survey on Unused or Expired Household Medications Disposal in Europe

Survey Objectives

The aim of this survey is to provide a comprehensive snapshot of national legislation, current practices, and the economic aspects related to the disposal of UNUSED OR EXPIRED HOUSEHOLD MEDICATIONS across EU member states. It aims to gather insights from European experts in healthcare, health policy, pharmaceutical regulation, and related fields to explore regional differences, identify best practices, and assess the ecological and economic impacts associated with medical waste related to e.g. medication non-adherence. The findings will contribute to a scientific publication that presents results from across Europe and supports the formulation of evidence-based policy recommendations to address these pressing issues.

Respondents' Benefits
National experts who provide a timely and comprehensive set of information about their country will be credited as 'collaborators' in the target publication. This acknowledgment ensures their contribution is searchable in scientific databases like PubMed.

Instructions for Completion
Please complete the survey based on the most recent available data for your country.
If exact data is not available, provide estimates or indicate “Data Not Available.”

For DISPOSAL Study Team,
Przemyslaw Kardas & Tamás Ágh

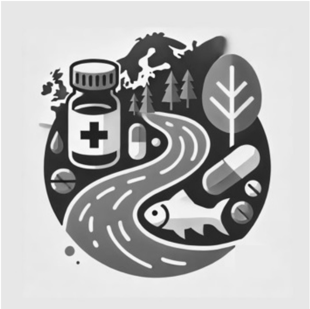

National Expert Survey on Unused or Expired Household Medications Disposal in Europe

Section 1: General Information

1. What is your country of residence?

2. What is your primary area of expertise?

- ○ Healthcare/pharmacy
- ○ Environmental policy
- ○ Waste management
- ○ Pharmaceutical regulation
- ○ Other (please specify)

3. How many years of professional experience do you have in related fields?

- ○ Less than 5 years
- ○ 5–10 years
- ○ More than 10 years

4. What is your primary affiliation type?

- ○ Government agency
- ○ Academic institution
- ○ Pharma company/other industry
- ○ Non-Governmental Organization (NGO)
- ○ Other (please specify)

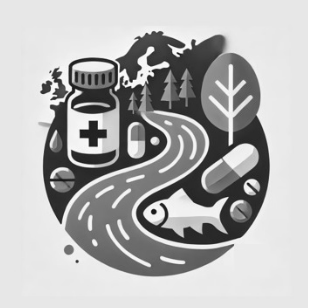

National Expert Survey on Unused or Expired Household Medications Disposal in Europe

Section 2: Legislative Framework for Collection of Unused or Expired Household Medications

5. Is there any national legislation that specifically addresses the collection of unused or expired household medications?

- ○ National Waste Act or equivalent regulations
- ○ Obligations established under Extended Producer Responsibility (EPR) scheme
- ○ Specific decrees or laws recognizing unused or expired household medications as hazardous waste and regulating their management
- ○ Municipal rules for waste handling
- ○ No specific legislation in place
- ○ Other (please specify)

6. Please provide a short description of the principal national legislation addressing the collection of unused or expired household medications, along with the year of enactment and a link to the official document of this legislation.

7. If organised medication collection is taking place, can patients provide unused or expired drugs for collection anonymously?

- ○ Yes
- ○ No
- ○ Not applicable
- ○ Other (please specify)

8. Are there any penalties for citizens for the improper disposal of unused or expired medications (e.g., flushing, burning, or landfilling)?

- ◯ Yes
- ◯ No
- ◯ Not applicable
- ◯ Other (please specify)

9. Please provide a short description of these penalties, if applicable

10. Do drugs distributed in your country typically include information about proper disposal of unused or expired drugs on the packaging and/or in the patient information leaflet?

- ◯ Yes
- ◯ No
- ◯ Other (please specify)

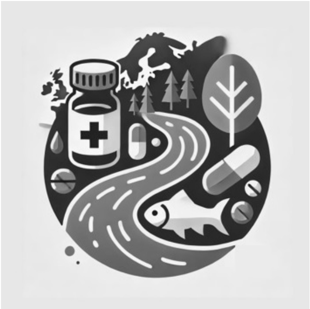

National Expert Survey on Unused or Expired Household Medications Disposal in Europe

Section 3: National Collection Program of Unused or Expired Household Medications

11. Is there any special system in place for the collection of unused or expired household medications in your country?

- ○ National program
- ○ Regional program(s)
- ○ Local program(s) (e.g. municipal system)
- ○ Lack of such a program
- ○ Other (please specify)

[ ]

12. What is the core principle of this program? Choose the one you consider most important.

- ○ Obligatory collection by pharmacies: pharmacies are legally obliged to collect unused or expired drugs from the public
- ○ Voluntary collection by pharmacies: pharmacies may choose to collect unused or expired drugs but are not legally required to do so
- ○ National Extended Producer Responsibility (EPR) scheme: a dedicated organization, appointed by the authorities, to manage the collection of unused or expired drugs
- ○ Return-to-manufacturer scheme: a program where unused or expired drugs are returned to the pharmaceutical manufacturer for disposal
- ○ Legally recommended disposal of unused or expired drugs in household (municipal) waste: authorities recommend disposing of unused or expired drugs in regular trash bins
- ○ Legally recommended disposal of unused or expired drugs in sewage/sink/toilet: authorities recommend flushing or pouring unused or expired drugs into the sewage system
- ○ Other (please specify)

[ ]

13. Please provide a short description of this program (how it operates, and its key features) and include a link to the program's official website or provide references to any relevant publication, if available

[ ]

14. Is the reuse of collected medications (e.g., redistributing unused, unexpired drugs to those in need) a common practice in your country?

- ◯ Not at all, it is forbidden by the law
- ◯ It is infrequent
- ◯ Neither frequent or infrequent
- ◯ It is frequent
- ◯ Not applicable (collection does not take place)
- ◯ Other (please specify)

15. Is there any special collection program in your country for the safe disposal of used prefilled syringes, injectors etc. (e.g., empty injection pens, insulin pens, adrenaline injectors, slimming drug injectors etc.)?

- ◯ National program
- ◯ Regional program(s)
- ◯ Local program(s) (e.g. municipal system)
- ◯ Lack of such a program
- ◯ Other (please specify)

16. Please provide a short description of this program (how it operates, and its key features) and include a link to the program's official website or provide references to any relevant publication, if available

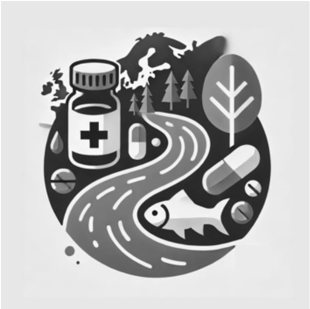

National Expert Survey on Unused or Expired Household Medications Disposal in Europe

Section 4: National Practices for Collection of Unused or Expired Household Medications

17. What is the most common practice for collecting of unused or expired household medications in your country?

- ◯ Pharmacies as primary collection points (pharmacy take-back programs)
- ◯ Collection at pharmacies AND other healthcare institutions (e.g., hospitals, healthcare centers, clinics etc.)
- ◯ Designated disposal points in municipal areas
- ◯ Public/municipal collection centers
- ◯ Mobile collection centers
- ◯ Hazardous waste collection points
- ◯ Special collection events
- ◯ Disposing of them in regular household waste (standard waste collection with household rubbish)
- ◯ Flushing them down the toilet or sink
- ◯ Other (please specify)

____

18. What is the final disposal method for collected unused or expired medications? Choose the one(s) you consider most important.

- ☐ Incineration
- ☐ Landfill
- ☐ Recycling (i.e. redistributing collected unused not expired drugs to those in need)
- ☐ Not applicable - unused or expired drugs are not collected
- ☐ Other (please specify)

____

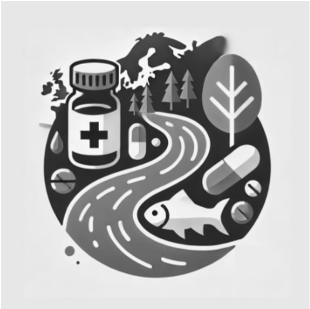

National Expert Survey on Unused or Expired Household Medications Disposal in Europe

Section 5: Public Awareness

19. In the last five years, have any national or regional public awareness campaigns been organized in your country promoting the proper disposal of unused or expired medications?

- ○ Yes
- ○ No
- ○ Other (please specify)

[ ]

20. If yes, please provide examples of these campaigns, including their key messages, as well as a link to its official website, if available.

[ ]

21. Have any of these campaigns been evaluated for effectiveness?

- ○ Yes
- ○ No
- ○ Not applicable (no campaigns have been organized in the past five years)
- ○ Other (please specify)

[ ]

22. If yes, please provide references to any relevant publications or a link, if available.

[ ]

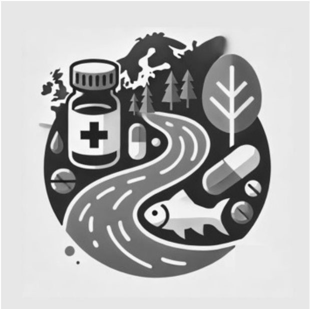

National Expert Survey on Unused or Expired Household Medications Disposal in Europe

Section 6: Economic Dimensions of Unused or Expired Household Drug Disposal

23. Who is responsible for covering the costs of collecting of unused or expired household medications in your country?

- ◯ National government
- ◯ Local government/municipalities
- ◯ Healthcare system
- ◯ Pharmacies
- ◯ Manufacturers under Extended Producer Responsibility (EPR) scheme
- ◯ Distributors / Wholesalers
- ◯ Collaborative funding by public and private entities (e.g. costs split between producers and municipalities; pharmacies and municipalities).
- ◯ Not applicable
- ◯ Other (please specify)

24. If pharmacies collect unused or expired household medications, are they reimbursed for the associated costs?

- ◯ Yes
- ◯ No
- ◯ Not applicable
- ◯ Other (please specify)

25. Who is responsible for covering the costs of the final disposal of collected unused or expired medications?

- Government
- Local government/municipalities
- Pharmacies
- Healthcare system
- Manufacturers
- Distributors/Wholesalers
- Not applicable (no collection takes place)
- Other (please specify)

26. What is the estimated annual volume of unused or expired household medications in your country (in kilograms/tons, if available)? Please provide reporting year.

Prescription drugs

Over-the-counter (OTC) drugs

All drugs

27. What is the average annual spending per person in your country on (in euro, if other currency used, recalculate to euro and include the exchange rate. Please provide reporting year):

Prescription drugs

Over-the-counter (OTC) drugs

All drugs

28. What is the annual per capita collection rate of household pharmaceutical waste in your country (if data available)? Please provide reporting year)

29. Based on available data, please provide the ATC codes (or API - Active Pharmaceutical Ingredient - names, if ATC codes are not available) of the top 10 most frequently wasted/collected unused or expired drugs in your country.

1 [ ]

2 [ ]

3 [ ]

4 [ ]

5 [ ]

6 [ ]

7 [ ]

8 [ ]

9 [ ]

10 [ ]

Please provide source and reporting year [ ]

30. Are national statistics available on the reasons for household drug wasted (e.g., expired drugs, medication non-adherence, etc.)?

- ◯ Yes
- ◯ No
- ◯ Not applicable
- ◯ Other (please specify)

[ ]

31. If yes, please summarize the key findings, provide the source and reporting year.

[ ]

32. What is the estimated annual cost of collecting and disposing of unused or expired household medications in your country? Please provide a source and reporting year

[ ]

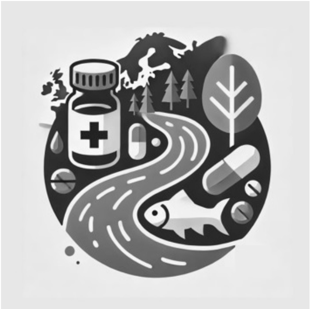

National Expert Survey on Unused or Expired Household Medications Disposal in Europe

Section 6: Additional Comments

33. Are there any unique challenges related to unused or expired household drug disposal in your country that you would like to share?

34. Are there any unique innovative solutions related to unused or expired household drug disposal in your country that you would like to share?

35. Do you have any additional data, reports, or case studies that could support this research?

36. In order to let you be included in the contributors' list of the target publication(s), we need your details. Please provide them here below:

Name

Surname

e-mail address

Affiliation (the way you want it to appear in target publication)

ORCID Number
